# Specific Extracellular Matrix Remodeling Signature of Colon Hepatic Metastases

**DOI:** 10.1371/journal.pone.0074599

**Published:** 2013-09-04

**Authors:** Maguy Del Rio, Caroline Mollevi, Nadia Vezzio-Vie, Frédéric Bibeau, Marc Ychou, Pierre Martineau

**Affiliations:** 1 IRCM, Institut de Recherche en Cancérologie de Montpellier, Montpellier, France; 2 INSERM, U896, Montpellier, France; 3 Université Montpellier1, Montpellier, France; 4 ICM, Institut régional du Cancer Montpellier, Montpellier, France; The University of Hong Kong, China

## Abstract

To identify genes implicated in metastatic colonization of the liver in colorectal cancer, we collected pairs of primary tumors and hepatic metastases before chemotherapy in 13 patients. We compared mRNA expression in the pairs of patients to identify genes deregulated during metastatic evolution. We then validated the identified genes using data obtained by different groups. The 33-gene signature was able to classify 87% of hepatic metastases, 98% of primary tumors, 97% of normal colon mucosa, and 95% of normal liver tissues in six datasets obtained using five different microarray platforms. The identified genes are specific to colon cancer and hepatic metastases since other metastatic locations and hepatic metastases originating from breast cancer were not classified by the signature. Gene Ontology term analysis showed that 50% of the genes are implicated in extracellular matrix remodeling, and more precisely in cell adhesion, extracellular matrix organization and angiogenesis. Because of the high efficiency of the signature to classify colon hepatic metastases, the identified genes represent promising targets to develop new therapies that will specifically affect hepatic metastasis microenvironment.

## Introduction

Colorectal cancer (CRC) is the third most common cancer in the world with 1.2 million new cases and more than 600,000 deaths every year [1]. In CRC, about 40% of patients will develop metastases. Because the venous drainage of the colon is through the portal vein, which goes directly to the liver, more than 70% of the CRC metastases are located in the hepatic tissue. In about 50% of the metastatic patients this is the unique metastatic location. Metastatic evolution results in a very poor prognosis with a median survival of about two years in treated patients. Long term survival is however possible in the 15% of patients that can benefit of hepatic metastasis surgery, usually after induction chemotherapy [2]. Improvement of current chemotherapies of CRC liver metastases will result in a higher proportion of patient benefiting from surgical resection, in longer survival time and ultimately in a higher proportion of cured patients.

Metastasis dissemination is a multi-step phenomenon still not completely understood [3]. For distant dissemination, a cell must first evade the primary tumor site and access venous or lymphatic circulation. This isolated cell must survive in the blood or lymphatic stream until it reaches a new organ where it will stop and adhere to endothelial cells in the capillary beds. Extravasation from the vessels into the organ will then eventually take place and cell will finally establish itself as a tumor by invasion and proliferation, recruiting stromal cells and building a new vascular network. Numerous genes are presumably implicated in these processes but are not fully identified yet. A better understanding of these mechanisms should allow to develop new therapeutic treatments that could target each of these steps. In current clinical practice, several adjuvant therapies are able to decrease metastatic dissemination. In CRC, oxaliplatin/5FU combined therapy significantly increases disease free and overall survival in stage III patients and thus decreases metastasis development [4]. However, such a therapy targets cell proliferation and not directly the metastatic process.

Few studies compared microarray data from primary colon tumors and metastatic tissues to identify genes implicated in cancer progression [5]. Three studies focused on the development of diagnostic and prognostic markers and did not try to identify gene signatures able to differentiate metastatic from primary cancer tissues [6]–[8]. Two studies presented gene signatures associated with metastatic disease containing more than 400 genes. Such long lists of genes are difficult to use for the development of new therapies [9], [10]. Among the five studies aimed at identifying the molecular mechanisms taking place during metastatic dissemination and growth, two did not succeed in the identification of a signature able to clearly separate primary cancers from metastatic tissues. In these two latter studies, authors analyzed pairs of primary and metastatic tumors and showed that samples clustered by patient and not by tissue origin [11], [12]. This suggests that heterogeneity between patients is higher than between a primary tumor and its metastases. Finally, the three gene signatures published so far [9], [13], [14] share only few genes [8], underlining the difficulty of extracting pertinent data from the background due to human diversity, cancer heterogeneity and the use of different microarray platforms.

Because of the difficulty of getting a robust signature from clinical samples, several authors have used model cell lines to identify genes associated with metastatic dissemination [9],[15],[16]. However, if working with cell lines will solve the problem of inter-individual variations, tissues and corresponding cell lines have different gene expression profiles [17]. This questioned the validity of a cell line based approach for clinical applications except when the results were crossed with those obtained with patient samples [16].

Another approach to remove the noise due to inter-individual variations is to use paired samples of primary and metastatic tissues in a homogeneous group of patients. Proper statistical test for paired samples allows the identification of genes implicated in the unique difference between the tissues, the metastatic location versus the primary tumor site. However, collection of such paired tissues is difficult since most of the metastases are not surgically removed. In addition, surgery of metastases takes place after chemotherapy, which presumably modifies metastasis expression profiles. This explains why only five studies have used such paired samples in CRC patients [7], [11]–[14]. Two of these studies did not succeed in the identification of a gene signature able to separate primary tumors from metastases [11], [12] and two studies did not try to identify such a signature [7], [14]. Only Ki et al [13] reported a 46-gene signature that perfectly separated the two classes. The signature was able to correctly classify 15 out of 18 samples of a training set. This training set was however collected in the same study and an external validation of this signature is still lacking.

To identify genes implicated in metastatic colonization of the liver in CRC, we collected pairs of primary tumors and hepatic metastases before chemotherapy in a homogeneous group of 13 patients. We compared mRNA expression in the pairs of patients to identify genes deregulated during metastatic evolution. We then validated the identified genes using public data obtained by different groups using different array platforms. By crossing the results obtained on different platforms and in related but different clinical models we sought to identify a gene signature robust enough to reliably point out to common mechanisms that may be targeted in patients.

## Materials and Methods

### Ethics Statement

The study was approved by ICM (Institut du Cancer de Montpellier) CORT (Comité de Recherche Translationnelle) ethical committee and all participating patients were informed of the study and had to provide signed written informed consent before enrollment.

### Patients and Tissue samples

Forty colorectal cancer patients with synchronous and unresectable liver metastases were enrolled in a prospective study at the ICM Cancer center from January 2000 to June 2004 [18]. Normal colon, colon cancer and hepatic metastasis samples were collected at the time of surgery, prior to chemotherapy.

All tissue samples were identified with a two-letter code. The first letter identifies the tissue origin (C: Colon, H: Hepatic, L: Lung, P: Peritoneum) and the second letter the tissue tumor state (N: Normal, P: Polyp, T: primary Tumor, M: Metastasis).

### RNA preparation and hybridization

After RNA extraction using RNeasy® mini Kit (Qiagen), a small fraction of the total RNA preparation was taken to determine the quality of the sample and the yield of total RNA. Controls were performed by UV spectroscopy and analysis of total RNA profile using the Agilent RNA 6000 Nano LabChip® Kit with the Agilent 2100 Bioanalyser (Agilent Technologies, Palo Alto, CA) to determine RNA purity, quantity, and integrity.

First strand cDNA synthesis was generated using a T7-linked oligo-dT primer, followed by second strand synthesis. Labeled cRNA probes were then generated by reverse transcription followed by in vitro transcription, incorporating biotin labeling, as part of the standard Affymetrix protocol. For each sample, the probes were then hybridized to human genome U133A chips (Affymetrix Inc., Santa Clara, CA) containing over 22 000 qualifiers, corresponding to genes and expressed sequence tags (EST). Data acquisition, processing and normalization were done as previously described [18]. The microarray data were deposited in the public Gene Expression Omnibus (GEO) database (www.ncbi.nlm.nih.gov/geo/) under accession number GSE49355.

### Data analysis

All data analyses were done using the R statistical environment [19]. A two-class paired SAM was implemented, using the R package “samr” [20], in order to compare gene expression between CT and HM. SAM allowed the identification of genes whose expression varied significantly through the 26 paired samples. If False Discovery Rate (FDR)<0.001, gene expression was considered significantly different.

Hierarchical unsupervised clusterings were performed using the hcluster method of R package “amap” [21] and the plots using the heatmap.2 function of “gplots” package [22]. Gene and sample distances were calculated using absolute Pearson and Pearson distances respectively. Linkages were done using the Ward algorithm. Inter-study normalization used the Bioconductor package “inSilicoMerging” [23], [24] using an Empirical Bayes method [25]. Gene- and pathway-enrichment analyses were done using the DAVID web-server [26], [27] and ClueGO Cytoscape plugin [28], [29] using Gene Ontology release 28.03.2013_18h05 [30]. ClueGO parameters were: MinLevel  = 2; MaxLevel  = 10; NoGenes1  = 4; MinPercentage1 = 1.0; GOFusion  =  true; GOGroup  =  true; KappaScoreThreshold  = 0.5; GroupByKappaStat  =  true; Correction Method Used  =  Bonferroni step down.

## Results

From the 40 CRC patients with synchronous and unresectable liver metastases enrolled in this study, we obtained expression data for at least one tissue in 28 patients (Table S1). This represented 18 normal colon mucosas (CN)^*^, 20 colon primary tumors (CT) and 19 hepatic metastases (HM). Most of the primary tumors were located in the left colon and histological analysis showed a well or moderate differentiation state. Among those 57 analyzed tissues, both CT and HM were collected in 13 patients (Table 1). These 13 pairs of samples were used to identify genes differentially expressed in HM versus the corresponding CT.

**Table 1:** Clinical characteristics of patients included in the training set.

		N = 13	%
**Gender**	Male	7	53.8
	Female	6	46.2
**Age (year), median [range]**		57	[45–70]
**WHO performance status**	0	5	38.5
	1	8	61.5
**Tumor location**	Right colon	1	7.7
	Transverse colon	1	7.7
	Left colon	10	76.9
	Rectum-sigmoid junction	1	7.7
**Differentiation**	Well	6	46.2
	Moderate	6	46.2
	Poor	1	7.6
**Synchronous metastatic**	Yes	13	100
**pN**	pN0	3	23.0
	pN1	4	30.8
	pN2	6	46.2
**pT**	pN3	10	76.9
	pN4	3	23.1

### Identification of a specific gene signature

Expression profiling of the 26 samples was conducted on Affymetrix human U133A chips containing 22200 probes corresponding to about 12700 genes. We only considered the 12408 probes that were present in at least 50% of the samples. After normalization and log2 transformation, we identified genes differentially expressed between CT and HM using the SAM method and a paired t-test statistics. Based on a false discovery rate (FDR) of 0.1%, we obtained a first list of 66 probes.

A major problem with HM samples is that they may contain contaminant normal liver tissue. This may lead to the false identification of genes since liver and colon tissues have very different gene expression profiles [13]. To minimize this problem, HM samples were collected and microscopically checked by a pathologist. Only those containing at least 50% of cancer cells were retained in this study. This still however allowed a large contamination of the HM tissues with normal liver. We thus used a simple bioinformatics method to identify within the 66 probes those for which the variation between CT and HM cannot be explained by such a contamination. Let consider that a probe p gives the same signal in a pair of CT and HM tissues (pCT = pHM) and a different signal, pHN, in the paired normal hepatic tissue (HN). Since HM sample may contain some HN tissue, the measured pHMm is different from pHM and is 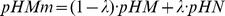, where λ is the fraction of HN contamination (0–0.5).

**Figure 1:**
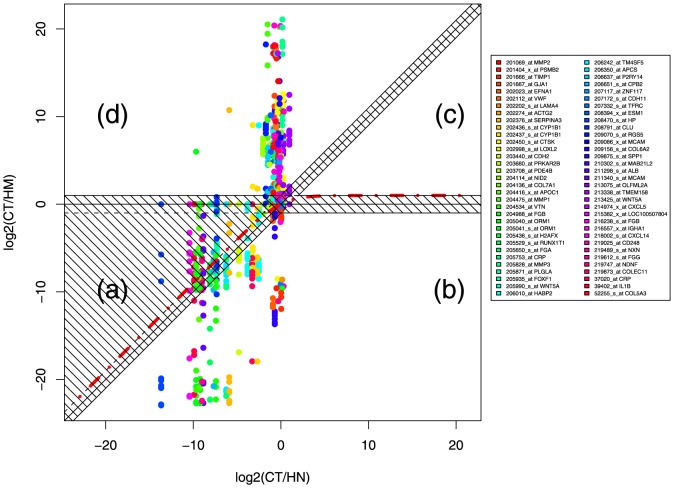
Identification of the 34-probe gene signature. The CT/HM values for the 13 pairs of samples and the 34 probes identified using paired SAM analysis were plotted versus the CT/HN values obtained in one paired sample from Sheffer's study [7]. The horizontal axis and the diagonal (HM = HN) separate the graph in four quadrants. (a) Genes over-expressed in HM versus CT, and downregulated in HM versus HN. (b) Genes over-expressed in HM versus CT, and in HM versus HN. (c) Genes downregulated in HM versus CT, and over-expressed in HM versus HN. (d) Genes downregulated in HM versus CT, and in HM versus HN. The red dashed line corresponds to a simulated contamination of a CT sample with 50% of a HN sample (see equation 1 in results section). The hatched region corresponds to HM samples that either are expressed at a comparable level (less than a 2-fold difference) in HM and CT or HM and HN, or whose variation between CT and HM can be explained by a contamination of HM by HN.

Thus the measured pCT/pHMm ratio is: 
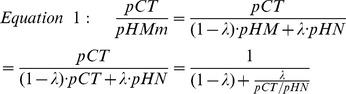



Since we did not collect paired HN samples in our study, we compared our measured pCT/pHMm values to the pCT/pHN ratios obtained from 6 paired patients previously analyzed using the same Affymetrix platform by Sheffer et al [7]. For each of the 66 probes identified in our study we calculated the minimum and maximum values of the pCT/pHN ratios obtained with the 6 pairs of Sheffer's study. We then considered that a probe is a false positive if it falls within the interval defined by equation 1 when λ varies between 0 and 0.5. The process is illustrated in Fig. 1 in the case of the minimal pCT/pHN ratios obtained with the 6 Sheffer's paired samples. Each color corresponds to a probe and each dot to a sample. For each probe the pCT/pHN ratio is constant and the 13 pCT/pHMm ratios obtained with our 13 pairs of patients are thus aligned on a vertical line. When there is no contamination (λ = 0), equation 1 corresponds to the x-axis. Simulation of a contamination of 50% gives the red dotted line that starts from the diagonal +1 and goes to the horizontal y = +1 line, when data are plotted using logarithmic scales. Any point between the x-axis and the red dotted line can thus be explained by a hepatic contamination and must be discarded. If we divide the plot in four quadrants separated by the diagonal and the x-axis, this essentially removed all the points in quadrant (a). The analysis was done for both the minimal and the maximal pCT/pHN ratios obtained with the 6 reference pairs and only samples that were selected with both reference ratios were kept.

In addition, we were only interested in genes that are expressed at a different level in CT and HM and we only kept the dots in Fig. 1 corresponding to a CT/HM ratio of at least two, that is the dots at a minimal distance of 1 from the x-axis. We also removed the dots close to the diagonal because we sought to isolate possible therapeutic targets that must be expressed at a different level in the metastasis and in the surrounding normal liver tissue.

In summary, these filters removed all the dots present in the hatched region in Fig. 1 for the 6 pCT/pHN ratios obtained from Sheffer's study. Quadrant (a) was totally removed by this approach and quadrant (c) was almost empty whatever the pCT/pHN reference ratio used. The two remaining quadrants (b) and (d) correspond to genes up- and down-regulated in HM versus CT respectively. Finally, for each probe, we counted the number of dots in each quadrant and we kept the probes for which at least 8 out of the 13 pairs were present in the same quadrant. This procedure suppressed genes known as expressed by normal hepatic tissue like albumin and gave us the final list of 34 probes (Table 2 & 3).

**Table 2:** Downregulated genes in hepatic metastases compared to the paired primary colon tumor.

Probe	Symbol	Gene name
205828_at	MMP3	matrix metallopeptidase 3 (stromelysin 1, progelatinase)
202274_at	ACTG2	actin, gamma 2, smooth muscle, enteric
219747_at	NDNF	neuron-derived neurotrophic factor
207172_s_at	CDH11	cadherin 11, type 2, OB-cadherin (osteoblast)
52255_s_at	COL5A3	collagen, type V, alpha 3
204136_at	COL7A1	collagen, type VII, alpha 1
218002_s_at	CXCL14	chemokine (C-X-C motif) ligand 14
214974_x_at	CXCL5	chemokine (C-X-C motif) ligand 5
208394_x_at	ESM1	endothelial cell-specific molecule 1
216557_x_at	IGHD	immunoglobulin heavy constant delta
	IGHG1	immunoglobulin heavy constant gamma 1 (G1m marker)
39402_at	IL1B	interleukin 1, beta
210302_s_at	MAB21L2	mab-21-like 2 (C. elegans)
209086_x_at	MCAM	melanoma cell adhesion molecule
213075_at	OLFML2A	olfactomedin-like 2A
206637_at	P2RY14	purinergic receptor P2Y, G-protein coupled, 14
203708_at	PDE4B	phosphodiesterase 4B, cAMP-specific
203680_at	PRKAR2B	protein kinase, cAMP-dependent, regulatory, type II, beta
201404_x_at	PSMB2	proteasome (prosome, macropain) subunit, beta type, 2
209070_s_at	RGS5	regulator of G-protein signaling 5
205529_s_at	RUNX1T1	runt-related transcription factor 1; translocated to, 1 (cyclin D-related)
215382_x_at	TPSAB1	tryptase alpha/beta 1
202112_at	VWF	von Willebrand factor
205990_s_at	WNT5A	wingless-type MMTV integration site family, member 5A
213425_at		
207117_at	ZNF117	zinc finger protein 117

**Table 3:** Upregulated genes in hepatic metastases compared to the paired primary colon tumor.

Probe	Symbol	Gene name
202437_s_at	CYP1B1	cytochrome P450, family 1, subfamily B, polypeptide 1
202436_s_at		
219873_at	COLEC11	collectin sub-family member 11
205753_at	CRP	C-reactive protein, pentraxin-related
204988_at	FGB	fibrinogen beta chain
206010_at	HABP2	hyaluronan binding protein 2
202376_at	SERPINA3	serpin peptidase inhibitor, clade A (alpha-1 antiproteinase, antitrypsin), member 3
209875_s_at	SPP1	secreted phosphoprotein 1
201666_at	TIMP1	TIMP metallopeptidase inhibitor 1

### External validations

**Figure 2:**
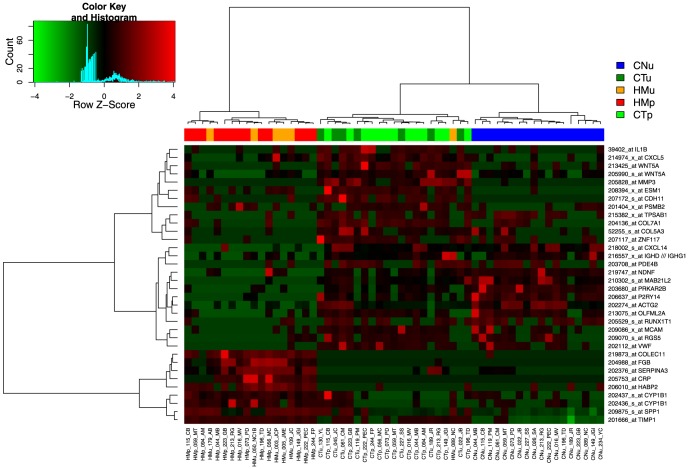
Hierarchical clustering of the samples collected in this study. HMp and CTp are the 13 paired samples used to identify the 34-probe signature (Table 1). HMu, CTu and CNu are additional samples collected in this study (Table S1).

The HM and CT samples used to derive the probe signature were perfectly separated by unsupervised hierarchical clustering (HMp and CTp in Fig. 2). This was however expected and we thus proceeded with external validations using different datasets.

During the collection of the paired samples, we collected and hybridized 6 HM and 7 CT additional unpaired samples. We also collected 18 normal colon (CN) tissues, among which 10 were from the same patients than the 13 paired CT and HM samples. We classified these 31 samples together with the 26 paired samples using two-way unsupervised hierarchical clustering. Three classes were clearly identified, one containing only HM samples, a second one with all the CT and one misclassified HM, and the last one containing all the CN tissues (Fig. 1 and Table 4). The unpaired and paired samples were mixed, showing that the gene expression pattern was not due to the paired nature of the training set. In addition, the 34-probe signature clearly discriminated CN from CT, demonstrating that the genes identified as deregulated during metastatic evolution were also modified during primary cancer formation. However, this validation set was of a modest size and we further validated our gene signature using external data available in public databases.

**Table 4:** External validation.

				Samples					
First author	Year	Platform	Nb genes 1	Type	Nb	Correct 2	Common genes 3	Data	Ref
This		Oligo	33	CT	7	7		GSE49355	
study		12,712		HM	6	5			
		Affymetrix		CN 4	18	18			
Sheffer	2009	Oligo	33	CT	186	183 5		E-GEOD-41258	[7]
		12,712		HM	47	42			
		Affymetrix		CN	54	52			
				CP	48	48 5			
				HN	13	13			
				LM	20	nc 6			
				LN	7	nc 6			
Ki	2007	cDNA	23	CT	47 7	46	CDH11	E-GEOD-6988	[13]
		17,104		HM	27	16	ESM1		
				CN	25	24	RGS5		
				HN	13	13	TIMP1		
							WNT5A		
Koh	2008	Oligo	25	CT	15 8	14	CRP	molpathol.org	[11]
		18,664		HM	12	12 9	CXCL14		
		Sigma		LM	3	2 9	CYP1B1		
				CN	15	15	IL1B		
							MMP3		
							SERPINA3		
							SPP1		
							WNT5A		
Kleivi	2007	Oligo	32	CT	18 10	17	None	Dr Lothe	[9]
		22,000		HM	4	4			
		Agilent		PM	4	nc			
Lin	2011	cDNA	24	CT	31	31	ACTG2	E-GEOD-22834	[8]
		19,500		HM	32	32	CDH11		
				HN	12	10	CYP1B1		
							ESM1		
							MCAM		
							RGS5		
							SERPINA3		
							SPP1 11		
							TIMP1		
							ZNF117		
All				CT	304	298	98%		
				HM	128	111	87%		
				CN	112	109	97%		
				HN	38	36	95%		

nc: non correctly classified

1 Number of the genes of our 33 gene signature present on the used platform.

2 Number of samples correctly classified using our signature (restricted to the number of genes in column “Nb genes”).

3 Genes in common between our 33 gene signature and those published in each study (Table 2 in Ki, Supplementary Table 2 in Koh, Fig. 2 in Kleivi, Supplementary Table 2 in Lin).

4 Ten of the CN samples are from the 13 paired patients used to identify the 34-probe signature.

**Figure 3:**
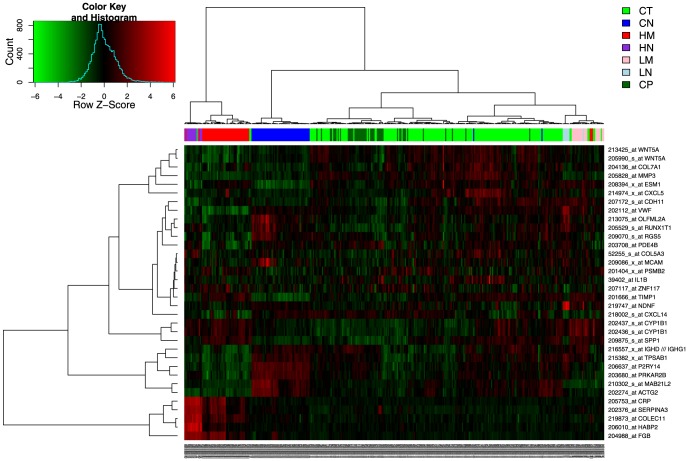
Hierarchical clustering of colon validation set. Data collected, hybridized and normalized by Sheffer et al. [7] were clustered using our 34-probe signature.

5 CT and CP are not clearly separated and are in a single class (Fig. 3).

6 LN and LM clustered in independent groups but were not separated from CT (Fig. 3).

7 27 CT with synchronous and 20 CT with metachronous metastases.

8 12 rectum and 3 colon tumors.

9 HM and LM are not separated and are in a single class.

10 8 right and 5 left colon tumors. 5 rectum tumors.

11 Only SPP1 is in the top 35 ranking genes in Lin's study (Fig. 1 in Lin).

The only publicly available dataset containing CT and HM samples of CRC using the same Affymetrix human U133A chips has been collected by Sheffer et al [7]. This large dataset contains not only CT and HM samples but also CN, HN, polyps (CP), lung metastases (LM) and normal lung tissues (LN). The 34-probe signature classified these samples in five main classes (Fig. 3 and Table 4). The first three classes corresponded to HN (13/13 HN, 1 CT and 2 HM), HM (42/47 HM and 2 CT), and CN (52/54) samples respectively. The two last classes regrouped most of the CP and CT samples (231/234), CP samples being mainly located in the fourth class (46/48). The last class also contained all the tissues extracted from the lung (LN and LM) in two sub-classes, one containing most of the LN (6/7) and the second one all the LM samples (20/20) with 7 CT and 3 HM samples. This clearly validated our probe signature since 183/186 (98%) CT and 42/47 HM (89%) were correctly separated. In addition, we also correctly classified CN samples, as previously shown in our validation set. HN samples were separated from HM tissues but 6 of them had been used in the filtering procedure and cannot be considered as an external validation set. However the 7 HN samples that were not used for the signature definition were also clustered independently of the HM samples, demonstrating the efficiency of our bioinformatics procedure to eliminate the signal due to a hepatic contamination.

### Stability to microarray technology

There is usually a good correlation between Affymetrix data and other techniques to measure mRNA abundance [31]. However, gene signatures are usually more platform dependent and must be validated to demonstrate their usefulness [32]. This is usually done by RT-PCR or immunohistochemistry but we alternatively used external data obtained in different laboratories using different array platforms.

A search for publicly available data identified four sets of CRC data containing HM and CT samples (Table 4). Two studies used home-made cDNA microarrays [8], [13], one used the Agilent human 1A platform [9], and the last one used home-spotted chips using the Sigma human Oligolibrary [11]. Because these platforms did not use Affymetrix probes, we first converted our probe signature into a gene signature. However, probe 216557_x_at corresponded to seven immunoglobulin genes. We thus looked in a larger list of 347 probes differentially expressed between CT and HM, according to the paired SAM analysis with a relaxed FDR of 10%, for specific probes for each of the seven genes associated with this 216557_x_at probe. We only found probes for IGHD and IGHG1 loci and we thus only used those two identifiers in the next analyses. The 34-probe signature was thus converted to a 33-gene signature (Table 2 & 3, column “Symbol”) because two genes were identified using two probes (WNT5A and CYP1B1) and the 216557_x_at probe was converted to two symbols (IGHD and IGHG1).

We used this 33-gene signature to classify the four datasets using unsupervised hierarchical clustering. However, those platforms contained only a subset of the 33 genes, from 32 in the best case down to 23 in the worst one (Table 4) and this may affect the quality of the obtained classifications. Despite this limitation, all the CT, HM, CN and HN samples were correctly classified with an accuracy of 83–100%, except in the case of Ki's study where only 16 out of 27 HM samples (59%) were correctly predicted (Table 4 and Fig. S1). This may be due to the small number of genes present in this last study since only 23 genes of our 33-gene signature were present in Ki's study and one gene (VWF) was absent in 34% of the samples (Fig. S1B). In addition, this data set contained a squamous cell carcinoma (SCC; sample DA09647) and a gastrointestinal stromal tumor (GIST; sample DX27754), both being misclassified as in the original published data.

### Conservation of gene variations

The clustering approach used in the previous results only showed that the 33 gene expression levels were modified between all the HM and CT samples, but the variation of these genes may be different in each dataset. It was not possible to compare the HM/CT ratios for all the genes in all the samples since many genes were absent in several studies, resulting in only four genes present in the six datasets.

We thus considered only the three datasets generated on commercial platforms because they contained all the genes but one of our signature. Two studies were done using Affymetrix human U133A chips (This study and Sheffer [7]) and the last one used the Agilent human 1A platform [9], which did not contain a probe for NDNF. To obtain reliable expression ratios, comparable between different platforms and studies, all the samples were renormalized altogether using a Empirical Bayes method [25]. The new normalized data allowed a clear separation of HM and CT using unsupervised hierarchical clustering since only 4 HM samples were incorrectly classified (Fig. S2A).

**Figure 4:**
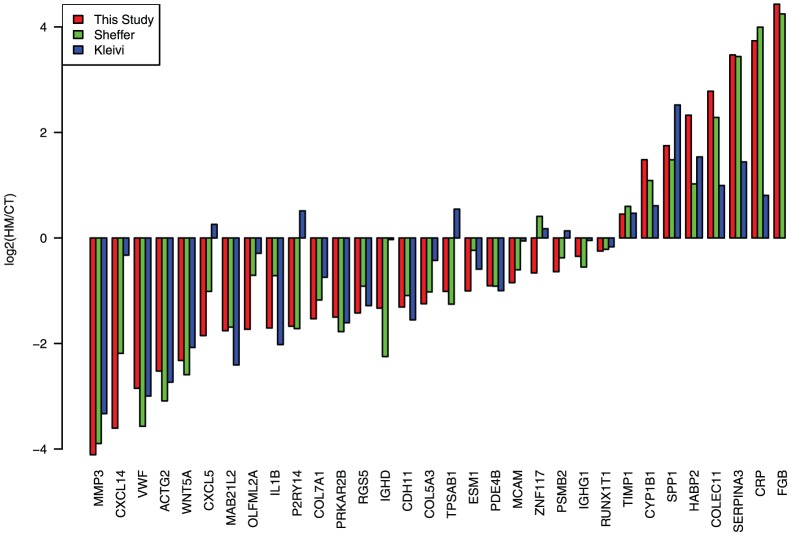
Expression of identified genes in three studies. Datasets from three intendant studies were renormalized together using an empirical Bayes method. Among the 33 genes of the gene signature, 32 genes were present in our (red), Sheffer (green) and Kleivi (blue) datasets (Table 4). The log2 ratio of HM to CT is plotted. Genes were ordered from the most downregulated to the most upregulated gene in HM versus CT in our study.

The common genes showed comparable expression modulation in the three datasets (Fig. 4). Not only they showed variations in the same direction but the HM/CT ratios were also well conserved, particularly in the two studies using the same Affymetrix platform. This showed that not only the 33 genes isolated are able to classify HM and CT samples but also that their variations are comparable in three independent studies.

### Specificity: metastatic location

CRC mainly forms distant metastases in the liver, but also in the Lung and in the peritoneal cavity. We asked whether our gene list was able to classify all the CRC metastases or if it was restricted to the hepatic location used to define the differentially expressed genes.

Three studies have collected CRC metastases located in another organ than liver: Sheffer [7] and Koh [11] collected several LM, and Kleivi some peritoneal carcinomatoses (PM) [9]. Using Affymetrix microarrays, Sheffer et al. analyzed 20 LM and seven LN tissues, which were partially classified using our gene signature. However, the classification was not as good as in the case of HM since they were not clearly separated from CT samples (Fig. 3). In addition, LM clustered in another branch than HM showing that if the deregulation of the 33 genes is partially conserved it is however different in the two metastatic locations. The small number of LM analyzed in the second study did not allow us to draw a clear conclusion, but the samples did not show any tendency to cluster together (Fig. S1A). The third study analyzed four CRC peritoneal carcinomatoses. Again it was difficult to draw a conclusion from such a small number of samples. Nonetheless, the four samples clustered neither together nor with HM by unsupervised hierarchical clustering using our gene signature (Table 4 and Fig. S1D). This demonstrated that the 33 genes identified are strongly associated with the hepatic location of the metastases.

### Specificity: primary tumor

We next examined the effect of the primary tumor origin to determine if the deregulated genes were specific to CRC.

We pooled five datasets of breast cancers originating from the same laboratory [33], [34]. The final set contained 198 primary breast cancers, 10 normal tissues, and metastases of different origins (4 in the liver, 13 in Lymph Nodes, 9 in the brain, 6 in the lung, and 10 from other locations). Unsupervised hierarchical clustering using our 34-probe signature did not show any tendency of any tissue type to form an independent cluster. Even the hepatic metastases did not cluster together showing that the identified genes are specific to CRC hepatic metastases (Fig. S3A). This also demonstrated the discriminating power of our approach to remove background signal due to metastatic contamination (Fig. 1). Indeed, if the identified signature was specific to liver tissue and not to CRC hepatic metastases, breast hepatic metastases should also cluster together.

This was further confirmed in squamous cell carcinomas of head and neck origin. None of the primary tumors, normal tissues and lymph node metastases collected in this study were separated using the 34-probe signature (Fig. S3B).

### Pathway enrichment analysis

**Figure 5:**
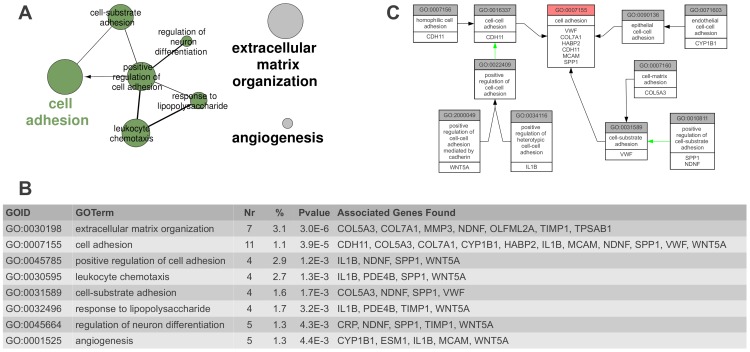
Functional annotation enrichment analysis of the 33-gene signature. Gene Ontology terms significantly over-represented in the 33-gene signature were identified and plotted using ClueGO. A) The size of the nodes are inversely proportional to the pvalue in Fig. 5B. Line widths between GO terms are proportional to the kappa scores used to define the categories. B) Table giving the results of the ClueGO analysis. Nr: Number of genes in our 33-gene signature associated with the GO term.%: Percentage of the genes of the considered GO term presents in our signature. Pvalue: pvalue of the GO term, corrected for multiple testing. C) Relation between the GO annotations of the 11 “cell adhesion”-associated genes in Fig. 5B and GO:0007155 term. Black arrows: “is a”. Green arrows: “Positively regulates”.

To get some insight on the pathways in which the 33 identified genes could be implicated, we performed gene- and pathway-annotation enrichment analyses using the DAVID web server and the Gene Ontology (GO) database. In this analysis we only used the IGHG1 gene symbol and not the the IGHD symbol associated to the same Affymetrix probe, resulting in a 32-gene signature. Only the KEGG pathway “ExtraCellular Matrix-receptor interaction” was significantly enriched with an EASE score (a modified Fisher Exact Pvalue) of 0.022 using DAVID web server. This was further confirmed by GO analysis using ClueGO software (Fig. 5). Eight GO terms were significantly enriched in our gene list. These eight terms were grouped in three pathways by ClueGO using kappa statistics, “Extracellular matrix organization”, “Cell adhesion” and “Angiogenesis”, which contained 50% (16/32) of the genes of the identified signature.

Altogether, our data show that it is possible to identify a short list of genes that differentiates primary tumors from hepatic metastases in CRC in six independent studies. The variation of these genes is well conserved, indicating a common mechanism that takes place during metastatic colonization of the liver by CRC cells.

## Discussion

To identify molecular mechanisms that regulate distant metastatic growth in CRC, we compared mRNA expression levels in paired primary tumor and hepatic metastatic tissues of 13 individuals. Despite the small size of the training set, we were able to define a 33-gene signature that discriminates 87% of HM from 98% of CT, 97% of CN and 95% of HN in six validation sets representing 128 HM, 304 CT, 112 CN and 38 HN samples and obtained on several commercial and home-made microarray platforms.

Global analysis of tissue samples must take into account that cancer tissues may contain a substantial proportion of the surrounding organ tissue. This is particularly true for small tumors and metastases. Comparison of the gene expression profiles of tumors located in two different organs may thus lead to the false identification of organ-specific genes instead of tumor-associated genes. This is particularly critical when the two organs have very different gene expression patterns, as it is the case of colon mucosa and liver [13]. We thus introduced a filtering step that removed the organ-specific genes and focus the signature towards metastasis and stroma associated genes. The efficiency of this filtering step is demonstrated by the external validations done in this study, since we correctly separated HM from HN tissues in three different studies (Fig. 3, Fig. S1B & C). In addition, if the identified signature was associated with the liver location of metastases, it should cluster together hepatic metastases whatever the location of the primary tumor. This is not the case since hepatic metastases of breast cancer were not grouped in the dendrogram (Fig. S3A). Finally, the signature clustered CN and CT tissues independently and is thus associated to some colon-specific modifications that originated in the primary tumor development. Other approaches have been previously used to solve the same issue. The best approach is to directly remove unwanted tissues by laser microdissection but it is time consuming and requires to work with a small number of cells. This has been done to study hepatic metastases of CRC in a small series of ten patients and led to the identification of genes mainly implicated in cell adhesion [14]. Ki et al. [13] developed a bioinformatics approach based on the first elimination of all the genes differentially expressed between normal colon mucosa and normal liver tissue before comparing hepatic metastases and primary tumors in CRC. As noted by the authors this may however lead to the elimination of important genes. Our approach is more conservative since the selected genes can be differentially expressed in normal liver and colon tissues.

The identified signature is independent of the stage of the primary tumor since two of the used validation sets contained stage I, II, III and IV cancers [7], [13]. In addition, stages did not cluster together and were randomly spread through the dendrogram (data not shown). This showed that the 33-gene expression pattern is constant in all primary tumors, independently of the presence of metastases. This is presumably because modulation of the pointed pathways takes place during metastatic migration and growth. In the primary tumors, those pathways are modulated only in the small subset of cells that will metastasize and are thus not detectable using global tissue analyses. Indeed, only a small subset of the primary tumor cells has the potency to migrate and form metastases, both in animal models [35] and in patients [36], and only a single cell analysis could identify them in the bulk of the primary tumor. Finally, the identified genes do not differentiate primary tumors according to their location. Because of the diversity of the datasets used in this analysis, primary tumors were located at several different locations in the colon and rectum (left and right colon, rectum, sigmoid, Table 4). Whatever the location, the efficiency of the 33-gene based clustering was the same. This is in agreement with large colorectal genome studies that showed that colon and rectal tumors are the same type of cancer [37]. The classification does not depend either on the MSI status or the p53 mutation state (data not shown). However, the gene signature failed to classify the hepatic metastases originating from a GIST and a SCC included in Ki's study. This was already the case in the original publication in which the authors showed that these two tumor types clustered independently from the other colorectal tumors. This is expected since most of the SCC are located in the lower portion of the rectum, often have mixed adenosquamous histologies and are more closely related to skin SCC than to CRC [38], and that GIST are sarcomas that originate in interstitial cells of cajal and rarely develop in colon.

The 33-gene signature identified is highly specific to primary colorectal tumors and their hepatic metastases. Indeed lung metastases of CRC were poorly classified and carcinomatoses did not cluster at all. Since dissemination of cancer cells through the portal vein is the same for lung and liver metastatic locations, it presumably means that several of the genes are important for long distance cell migration through blood vessels. This is not the case of carcinomatoses that often disseminate directly into the peritoneal cavity [39]. When applied to other cancer types, we were not able to separate metastases from primary tumors. This was even the case for hepatic metastases of breast cancer. The modulated pathways are thus specific both to the primary tumor type and to the metastatic location. This is in good agreement with the “seed and soil” hypothesis first formulated by Stephen Paget in 1889 [3], who proposed that metastasis growth depends on a cross-talk between a cancer cell and the host organ. However, the fact that lung metastases of CRC were partially classified indicates that the identified genes not only depend on the primary tumor type and the location of the metastases, but also on the dissemination pathway. This may explain the presence of several genes implicated in platelet activation and blood coagulation (TIMP1, VWF, PRKAR2B, FGB). Fibrinogen beta (FGB) was the most over-expressed gene in hepatic metastases and the von Willebrand factor (VWF) the second downregulated one in three independent studies (Fig. 4). In addition to their roles in blood coagulation, both genes have been previously implicated in metastasis dissemination. For instance, FBG [40] and VWF [41] deficiency respectively decreases and increases the metastatic potential of a lung carcinoma and a melanoma in mouse models. These effects have been demonstrated in knock-out mouse models and they may take place at any step from cell injection to metastasis formation in the lung. Since in our analysis the expression of these two genes is specifically modulated within the metastases, this argues in favor of a role of these genes in the last steps of metastasis formation.

Enrichment analysis showed that an unique KEGG pathway, ECM-receptor interaction, was significantly modulated in the hepatic metastases. In addition, most GO terms significantly enriched in our gene list are related to extracellular matrix adhesion and remodeling (Fig. 5). The GO Biological Process containing the largest number of genes is cell adhesion (GO:0007155). Six of the genes are indeed part of this GO Biological Process (VWF, COL7A1, HABP2, CDH11, MCAM, SPP1). In addition, four other genes (SPP1, IL1B, NDNF, WNT5A) are positive regulators of cell adhesion (GO:0022409 and GO:0010811), one (CYP1A1) is implicated in endothelial cell-cell adhesion (GO:0071603), and a last one (COL5A3) in cell-matrix adhesion (GO:007160) (Fig. 5C). This represents 11 genes (33%) of the identified signature. Most of these genes are downregulated in hepatic metastases (Fig. 4), resulting in a lower adhesion potential of the metastatic cell. The low-adhesion propensity of metastatic cells has been frequently observed and is presumably required for cell dissemination [42]. However two proteins implicated in cell adhesion are over-expressed in hepatic metastases, HABP2 and CYP1B1. HABP2 binds hyaluronate, one of the five constituents of the ECM matrix and has also an anti-angiogenic function [43]. Its expression level is however moderate in hepatic metastases with only a two-fold increase compared to normal colon mucosa (Fig. S2B). CYP1B1 over-expression in cancer has been previously reported. Its downregulation using siRNA leads, in an endometrial carcinoma cell line, to a decrease in cell proliferation and invasion [44]. They also showed that CYP1B1 positively regulates MCAM expression. This seems to be different in colon metastases since we have concomitant over-expression of CYP1B1 and downregulation of MCAM. However, CYP1B1 is expressed in endothelial cells and its expression promotes angiogenesis in a non-cancer mouse model [45]. Together with VWF, which is downregulated in tumor endothelial cells of CRC [46], CYP1B1 over-expression could promote angiogenesis in colon hepatic metastases. This is further enhanced by the downregulation of WNT5A [47] and CXCL14 [48] which have both been shown to negatively regulate angiogenesis. However the expression of two other genes that positively regulate angiogenesis, ESM1 and IL1B, are also decreased in hepatic metastases. The expression of ESM1 is, nevertheless, only slightly decreased in HM and still much higher than in normal colon and liver tissues, and the expression of IL1B is not strongly modified (Figs. 4 and S2B).

In addition to a decrease in ECM adhesion, a matrix metalloproteinase (MMP), MMP3, is strongly downregulated in hepatic metastases. Various proteinases are implicated in ECM turnover, but MMPs are the principal ECM-degrading agents. Several studies have shown that MMP, including MMP3, over-expression correlates with poor prognosis and metastatic evolution in several cancers, including colon adenocarcinomas [49]. In particular, colon metastases are known to induce MMP2 and MMP9 expression in stromal cells, and MMP inhibitors reduce tumor growth and metastasis in animal models [50]. This is thus particularly surprising to identify MMP3 as the most downregulated gene in hepatic metastases. Since this is the case in three independent studies (Fig. 4) this shows that it is not coincidental but corresponds to the metastatic evolution of colon adenocarcinoma. The most likely explanation is that MMP3 is required in the primary tumor, but no more needed once macroscopic metastases are established in the liver. This may explains why MMP3 expression strongly increased in colon adenocarcinoma and decreased back to a normal level in metastatic samples (Fig. S2B).

Despite several advances in the treatment of colorectal hepatic metastases, there is still a need of new targets to improve patient outcome. The 33 genes identified in this study have interesting properties that may make them promising therapeutic targets. First, we demonstrated that their deregulations are widely conserved in six different studies (Table 4). Second, they showed conserved levels of deregulation in three genome-wide studies (Fig. 4). Third, several of them were found in the signatures published so far (column “Common genes” in Table 4). Four, because these genes are specifically deregulated in hepatic metastases of CRC, targeting them may result in highly specific therapeutics. Five, many of these genes have already been shown to block or enhance metastatic potential either in cell lines (CYP1B1 [51]) or in animal models (VWF [41], FBG [40], SPP1 [52], WNT5a [53]). Finally, several of the identified proteins could be targeted either with chemicals or antibodies. The identified genes may be implicated in any of the necessary steps required to form a metastasis, from primary tumor escape to growth at the secondary site. If a gene is required for an early step and no more for the maintenance of macroscopic metastases in the liver, there is no reason for this gene to be conserved in 90% of the samples as demonstrated here. This argues in favor of a strong requirement of these genes for macroscopic metastasis survival in the liver, and thus for a possibility to affect metastases by targeting them. As it is already available in clinics with the anti-VEGF Bevacizumab that targets angiogenesis and thus the metastatic niche of colorectal liver metastases, some of the identified genes may offer opportunities to remodel the tumor environment, increase chemotherapy efficiency and improve patient survival in CRC.

## Supporting Information

Figure S1 **Two-way hierarchical clustering of colorectal cancer datasets.** All data were processed and normalized by the original authors. A) Normal colon tissues (blue), primary tumors (green), and hepatic (red) and lung (orange) metastases were clustered using the 25 genes of our 33-gene signature present in Koh et al. Study [11]. B) Normal colon (blue) and normal liver (light blue) tissues, primary tumors in metastatic (green) and non-metastatic (light green) patients, and hepatic (red) metastases were clustered using the 23 genes of our 33-gene signature present in Ki et al. Study [13]. The red and green bars bellow the heatmap indicate the hepatic metastasis samples from the GIST and the SCC respectively. C) Normal liver tissues (light blue), primary tumors (green) and hepatic metastases (red) were clustered using the 24 genes of our 33-gene signature present in Lin et al. [8] study. D) Normal colon tissues (blue), primary tumors (green), hepatic metastases (red) and peritoneal carcinomatosis (orange) were clustered using the 32 genes of our 33-gene signature present in Kleivi et al. [9] study.
(PDF)

Figure S2 **Gene expression variation in three independent studies.** Data collected in this study, in Sheffer et al [7]. and in Kleivi et al [9]. were renormalized together using an empirical Bayes method (Fig. 4). A) Normalized HM and CT samples were clustered using the 32 common genes. B) Boxplots of the expression levels of the 32 common genes in CN (blue), CT (green), HM (red) and HN (dark blue) tissues are plotted.
(PDF)

Figure S3 **Two-way hierarchical clustering of non-colorectal cancers and their metastases.** A) 198 primary breast cancers, 10 normal tissues, and metastases of different origins (4 in the liver, 13 in Lymph Nodes, 9 in the brain, 6 in the lung, and 10 from other locations). All samples were collected, analyzed and normalized in the same laboratory [33], [34]. Samples were clustered using the 22 genes of our 33-gene signature present in the study. Gene Expression Omnibus identifiers of the used datasets: GSE2740, GSE3521. B) Normal tissues (blue), primary tumors (green) and lymph node metastases (red) in head and neck squamous cell carcinomas. Samples were obtained from two sources, collected and normalized by Lukk et al. [17]. Samples were clustered using our 34-probe signature.
(PDF)

Table S1 Clinical characteristics of patients used in this study.
(PDF)
