# Supplementary figures and images for: Specific Extracellular Matrix Remodeling Signature of Colon Hepatic Metastases
Source: PLoS One. 2013 Sep 4;8(9):e74599. doi: 10.1371/journal.pone.0074599 (PMC3762755; doi:10.1371/journal.pone.0074599)

A

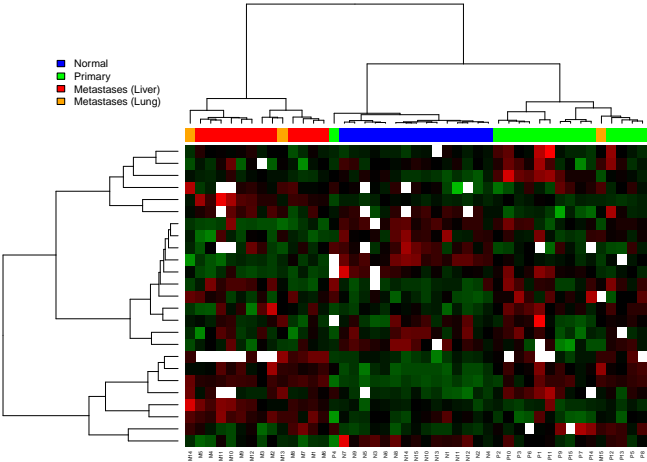

B

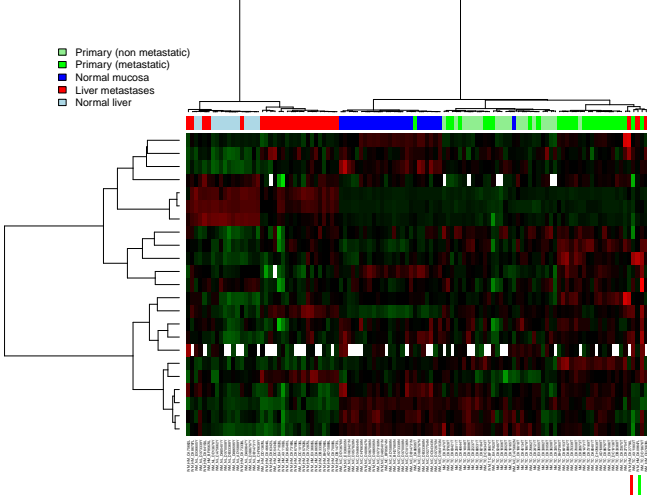

C

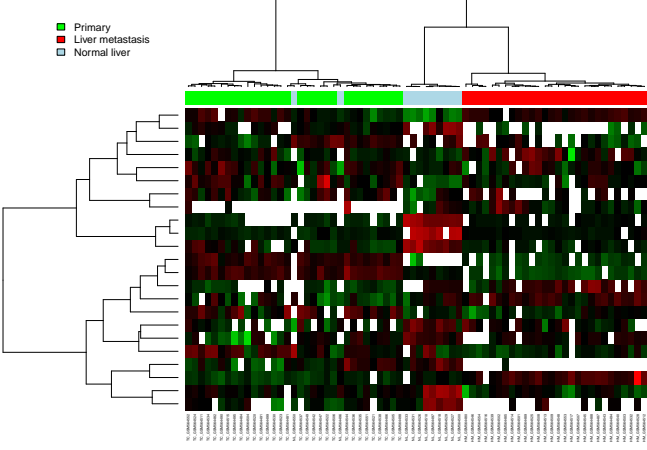

D

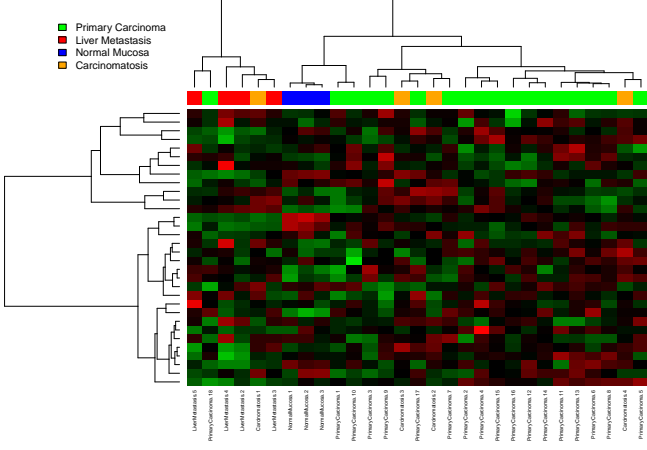

Supplement: Figure S1 — Two-way hierarchical clustering of colorectal cancer datasets. All data were processed and normalized by the original authors. A) Normal colon tissues (blue), primary tumors (green), and hepatic (red) and lung (orange) metastases were clustered using the 25 genes of our 33-gene signature present in Koh et al. Study [11]. B) Normal colon (blue) and normal liver (light blue) tissues, primary tumors in metastatic (green) and non-metastatic (light green) patients, and hepatic (red) metastases were clustered using the 23 genes of our 33-gene signature present in Ki et al. Study [13]. The red and green bars bellow the heatmap indicate the hepatic metastasis samples from the GIST and the SCC respectively. C) Normal liver tissues (light blue), primary tumors (green) and hepatic metastases (red) were clustered using the 24 genes of our 33-gene signature present in Lin et al. [8] study. D) Normal colon tissues (blue), primary tumors (green), hepatic metastases (red) and peritoneal carcinomatosis (orange) were clustered using the 32 genes of our 33-gene signature present in Kleivi et al. [9] study. (PDF) [file pone.0074599.s001.pdf]

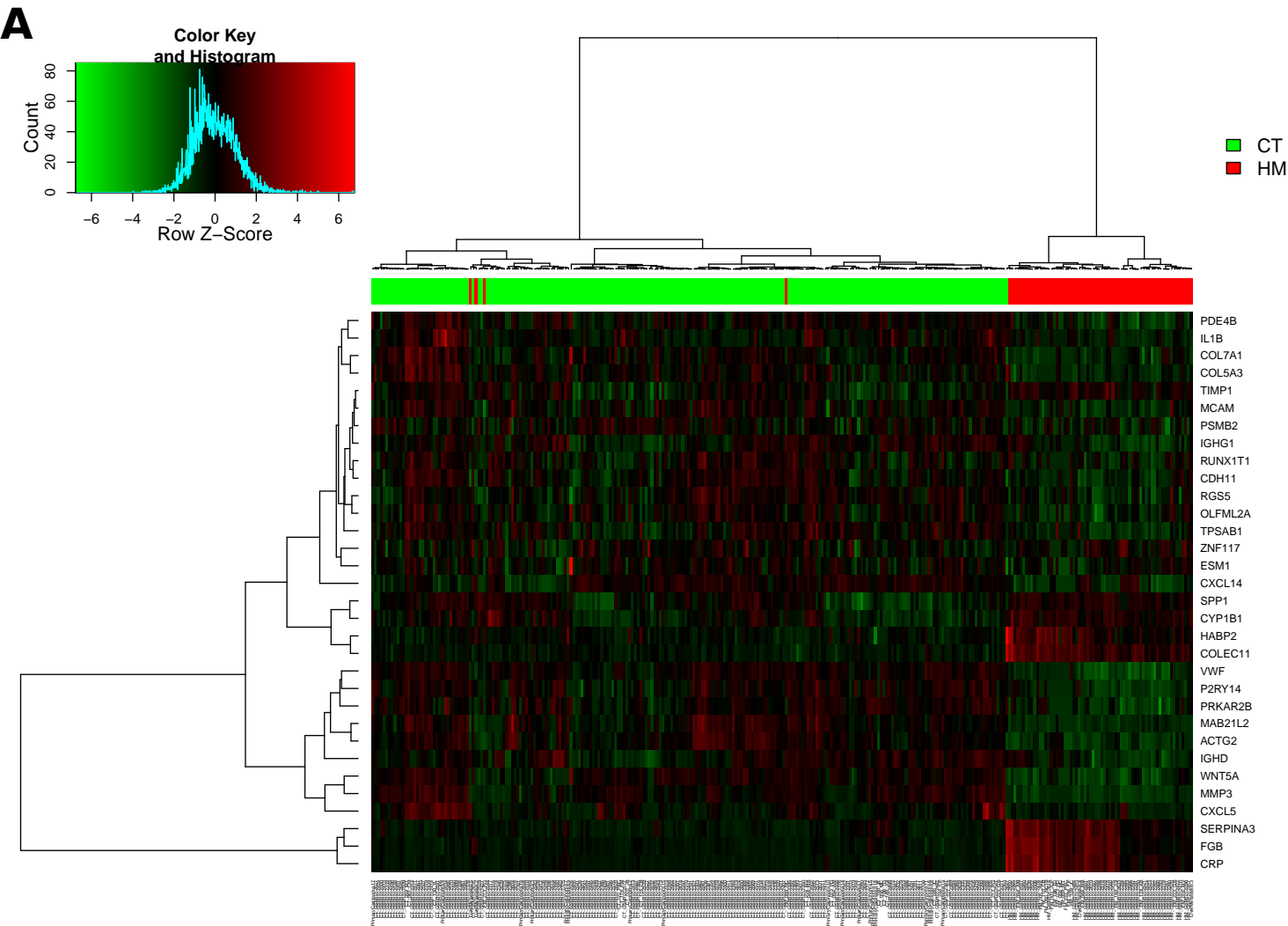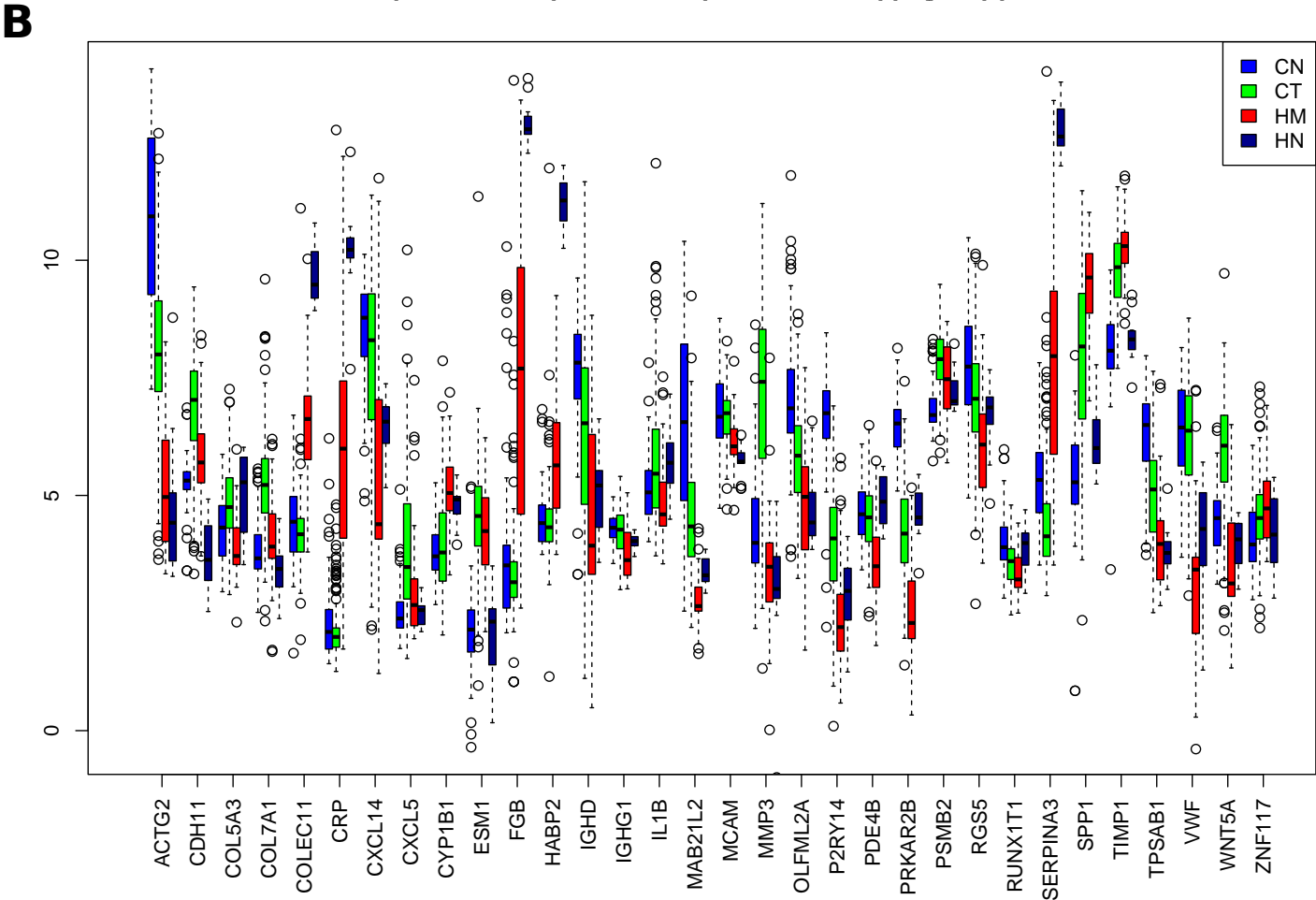

Supplement: Figure S2 — Gene expression variation in three independent studies. Data collected in this study, in Sheffer et al [7]. and in Kleivi et al [9]. were renormalized together using an empirical Bayes method (Fig. 4). A) Normalized HM and CT samples were clustered using the 32 common genes. B) Boxplots of the expression levels of the 32 common genes in CN (blue), CT (green), HM (red) and HN (dark blue) tissues are plotted. (PDF) [file pone.0074599.s002.pdf]
