# Supplementary material for: Specific Extracellular Matrix Remodeling Signature of Colon Hepatic Metastases
Source: PLoS One. 2013 Sep 4;8(9):e74599. doi: 10.1371/journal.pone.0074599 (PMC3762755; doi:10.1371/journal.pone.0074599)
Supplement: Figure S3 — Two-way hierarchical clustering of non-colorectal cancers and their metastases. A) 198 primary breast cancers, 10 normal tissues, and metastases of different origins (4 in the liver, 13 in Lymph Nodes, 9 in the brain, 6 in the lung, and 10 from other locations). All samples were collected, analyzed and normalized in the same laboratory [33], [34]. Samples were clustered using the 22 genes of our 33-gene signature present in the study. Gene Expression Omnibus identifiers of the used datasets: GSE2740, GSE3521. B) Normal tissues (blue), primary tumors (green) and lymph node metastases (red) in head and neck squamous cell carcinomas. Samples were obtained from two sources, collected and normalized by Lukk et al. [17]. Samples were clustered using our 34-probe signature. (PDF) [file pone.0074599.s003.pdf]

A

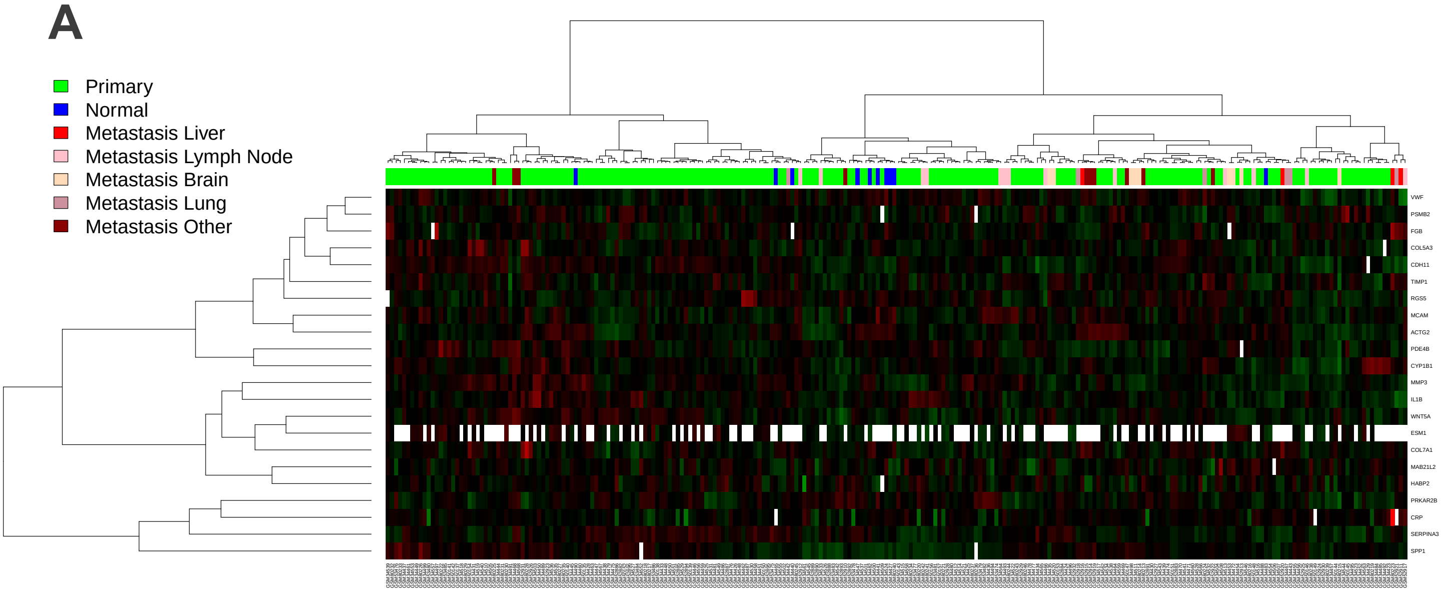
Primary
Normal
Metastasis Liver
Metastasis Lymph Node
Metastasis Brain
Metastasis Lung
Metastasis Other
VWF
PSMB2
FGB
COL5A3
CDH11
TIMP1
RGS5
MCAM
ACTG2
PDE4B
CYP1B1
MMP3
IL1B
WNT5A
ESM1
COL7A1
MAB21L2
HABP2
PRKAR2B
CRP
SERPINA3
SPP1

B

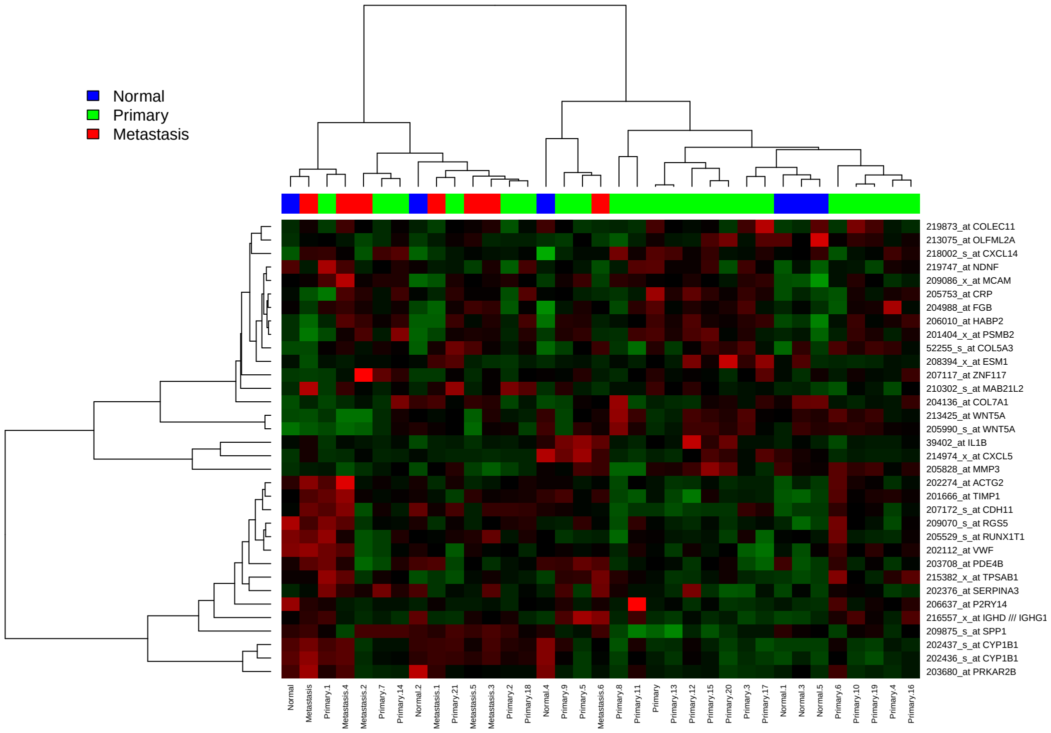
Normal
Primary
Metastasis
219873_at COLEC11
213075_at OLFML2A
218002_s_at CXCL14
219747_at NDNF
209086_x_at MCAM
205753_at CRP
204988_at FGB
206010_at HABP2
201404_x_at PSMB2
52255_s_at COL5A3
208394_x_at ESM1
207117_at ZNF117
210302_s_at MAB21L2
204136_at COL7A1
213425_at WNT5A
205990_s_at WNT5A
39402_at IL1B
214974_x_at CXCL5
205828_at MMP3
202274_at ACTG2
201666_at TIMP1
207172_s_at CDH11
209070_s_at RGS5
205529_s_at RUNX1T1
202112_at VWF
203708_at PDE4B
215382_x_at TPSAB1
202376_at SERPINA3
206637_at P2RY14
216557_x_at IGHD /// IGHG1
209875_s_at SPP1
202437_s_at CYP1B1
202436_s_at CYP1B1
203680_at PRKAR2B
Normal
Metastasis
Primary.1
Metastasis.4
Metastasis.2
Primary.7
Primary.14
Normal.2
Metastasis.1
Primary.21
Metastasis.5
Metastasis.3
Primary.2
Primary.18
Normal.4
Primary.9
Primary.5
Metastasis.6
Primary.8
Primary.11
Primary
Primary.13
Primary.12
Primary.15
Primary.20
Primary.3
Primary.17
Normal.1
Normal.3
Normal.5
Primary.6
Primary.10
Primary.19
Primary.4
Primary.16
