# Supplementary material for: Specific Extracellular Matrix Remodeling Signature of Colon Hepatic Metastases
Source: PLoS One. 2013 Sep 4;8(9):e74599. doi: 10.1371/journal.pone.0074599 (PMC3762755; doi:10.1371/journal.pone.0074599)
Supplement: Table S1 — Clinical characteristics of patients used in this study. (PDF) [file pone.0074599.s004.pdf]

Table S1. Clinical characteristics of patients used in this study

		N=28	%
**Gender**	Male	17	60.7
	Female	11	39.3
**Age(year), median[range]**		59.5	[45-76]
**WHO performance status**	0	14	53.8
	1	12	46.2
	Missing	2	
**Tumor localization**	Caecum	1	3.6
	Right colon	1	3.6
	Transverse colon	2	7.1
	Left colon	19	67.9
	Rectum-sigmoid junction	2	7.1
	Rectum	3	10.7
**Differentiation**	Well	11	45.8
	Moderate	10	41.7
	Poor	3	12.5
	Missing	4	
**Synchronous metastatic**	Yes	28	100
**pN**	pN0	6	22.2
	pN1	6	22.2
	pN2	15	55.6
	Missing	1	
**pT**	pN3	20	74.1
	pN4	7	25.9
	Missing	1	
